# Onions

**Published:** 1878-03

**Authors:** 


### ONIONS

Are one of the most nutritious, healthful, and detestable articles of food found in our markets. We never ate one to our knowledge, and never expect to; we can smell them a mile off, perhaps. A few grains of roasted coffee, eaten immediately afterwards, or a teaspoonful or two of vinegar swallowed, removes at once the odor from the breath. If onions are half-boiled, the water thrown away, and are then put in soup to be boiled "done," the odor will be but little noticed.

### A SAD SIGHT.

"It is one of the saddest in nature, to see the old spiteful and vindictive, querulous with all." Said a sunny-faced little girl to her mother one day :

"Mother, will grandfather go to heaven when he dies?"

"Yes, my child, I hope he will."

"Well, then I don't want to go there, for he is so cross."

Surely, time should mellow the heart of the aged, should fill it with sympathies, making it beam with forbearance towards all ; and thus it is with the guileless and the good. Those who are not so, we should pity ; for we may be sure they have missed the aim of life, have sown the seeds of wrong-doing in youth, which now, in their age, are bearing the wormwood and gall of the memory of ill-directed energies, of unjust purposes, of unholy ambitions, and it may be, of loving hearts, long ago wounded or broken by confidences falsified. Pity then, the malignant old man, who deals in the vile innuendo, drives a dagger from behind, or prostitutes a chance power, to feed his own hate; to such we say, be silent, pity and forgive.

---

### THE EMOTIONS.

We believe strongly in the emotions as an element of health and disease. Every person should strive to maintain an equable temper. Fretfulness and ill temper impair both moral and physical beauty. A scold bears that brand upon the countenance as indelible as the mark of Cain.

Good emotions, improve digestion, while the bad ones impair it; hence many of our articles have a direct bearing on health, although a dozen of them may be read without naming the words 'health,' 'disease,' 'doses,' or 'symptoms.' So, for "once and the last time," as the auctioneer says, we advertise our readers, that we can't be at the trouble to show what connection any particular subject we discuss has with health; they must work out the problem themselves, under the general rule laid down at the top of the first page of each number.

